# Study on the Genomic Basis of Adaptation in Salsk Sheep

**DOI:** 10.3390/biology14111620

**Published:** 2025-11-18

**Authors:** Olga Lukonina, Siroj Bakoev, Yury Kolosov, Vagif Akhmedli, Ilona Bakoeva, Maria Kolosova, Alexandr Usatov, Anatoliy Kolosov, Lyubov Getmantseva

**Affiliations:** 1 All Russian Research Institute of Animal Breeding, Lesnye Polyany 141212, Russia; lukoninaon@mail.ru (O.L.); siroj1@yandex.ru (S.B.); vaqifakhmedli205@mail.ru (V.A.); kolosov777@gmail.com (A.K.); 2 Biotechnological Faculty, Don State Agrarian University, Persianovsky 346493, Russia; kolosov-dgau@mail.ru; 3 Faculty of Physics, Mathematics and Natural Sciences, RUDN University: Peoples’ Friendship University of Russia, Moscow 117198, Russia; 4 Faculty of Biocybernetics and Systems Biology, Russian State Agrarian University—Moscow Agricultural Academy Named After K.A. Timiryazev, Moscow 127434, Russia; ilonaluba2@mail.ru; 5 Academy of Biology and Biotechnology Named After D.I. Ivanovsky, Southern Federal University, Rostov-on-Don 344006, Russia; usatova@mail.ru

**Keywords:** Salsk breed of sheep, selection signatures, adaptation, population genomics, candidate genes, iHS, nSL, iHH12, XP-EHH

## Abstract

This study investigates the genetic architecture of Salsk sheep—a long-established Russian Merino-type breed from the southern steppes—highlighting their broad genetic diversity, resilience to cold and drought, and dual-purpose (wool and meat) productivity as a unique gene pool shaped by natural and artificial selection. The study used data from 96 sheep. Genotyping was carried out on the Illumina Ovine Infinium^®^ HD BeadChip platform, and after filtering, 511,145 SNPs were retained. We assessed population structure and genetic diversity using principal component analysis (PCA), Fst, and linkage disequilibrium (LD) in comparison with four reference European breeds. To detect selection signatures, we employed a combination of complementary methods, including intra-population statistics (iHS, nSL, iHH12) and inter-population comparisons (XP-EHH). This integrated approach identified genomic regions under positive selection, reflecting the breed’s evolutionary response to both natural and artificial selection pressures. Strong selection signals were detected in genes associated with production traits like fertility and growth (*CCSER1*, *SOX6*), as well as fundamental adaptive functions, including immune response (*IL6R, NLRP1*) and energy metabolism (*ACSL5*, *FANCA*). These results elucidate the genetic basis of the Salsk breed’s high resilience and highlight its potential as a valuable genetic resource for improving this trait in other sheep populations.

## Simple Summary

The application of modern genomic methods provides key insights into how domestic animal breeds adapt to local conditions. The Salsk sheep breed, developed in the harsh continental climate of the Russian steppes, is renowned for its exceptional hardiness and productivity. This study aims to identify the fundamental genetic mechanisms underlying their adaptation to extreme steppe environments and diseases, as well as the genes under selection for key production traits such as fertility, growth rate, and wool quality. Understanding these genetic aspects is essential for the breed’s conservation and its strategic use in selective breeding programs.

## 1. Introduction

Merino sheep breeds are of significant economic importance in the Russian Federation. According to the Sheep and Goat Breeding Yearbook 2025 [1], the total sheep population in Russia reached 17.7 million heads, of which fine-wool breeds accounted for 57.9%. Historically, European Merinos were imported into Russia as early as the 18th–19th centuries. However, their demanding housing and feeding requirements prompted local breeders to develop better-adapted varieties. Among the first successful outcomes were the Russian Infantado, developed by I.A. Mertsalov, and the Mazayev breed, established by the Mazayev family [2]. Further progress in domestic Merino breeding was driven by Professor P.N. Kuleshov, who created the Novocaucasian Merino—a foundational breed for many modern lineages [2].

One of the most valuable domestic breeds is the Salsk sheep. Its development began in the 1920s in the Rostov region, marking a major achievement in breeding science. The breed was established by crossing local Merino ewes (Mazayev and Novocaucasian) with Rambouillet rams, resulting in robust animals with excellent conformation and high wool productivity [3]. By the time of its official recognition in 1950, the breed had been refined not only for wool quality and yield but also for key traits such as live weight, precocity, hardiness, and fertility.

Today, the Salsk breed is maintained at the Belozernoye stud farm (Rostov region), with ongoing efforts to enhance both its wool and meat production. The breed has been preserved in a limited number of farms, with approximately 2500 breeding ewes and around 200 rams currently registered. It is officially included in the State Register of Breeding Achievements of the Russian Federation and, although not under immediate threat of extinction, is classified as a local breed with a restricted population size that requires preservation of its genetic diversity. The Salsk sheep breed is characterized by high wool productivity (6–14 kg fleece yield with 45–55% clean fiber and fiber length of 7.5–8.5 cm), good reproductive performance (120–140 lambs per 100 ewes), and strong adaptation to the dry steppe climate [4]. Given the breed’s economic value and the need for its continued improvement, a comprehensive analysis of its genetic architecture to identify adaptive traits and loci under selection pressure is of critical importance.

Studying the genomic basis of local adaptation is essential for targeted breeding. Advances in genomics have enabled the identification of loci under positive selection through methods that scan the genome for characteristic signatures [5]. These signatures may arise from shifts in the allele frequency spectrum [6], inter-population differentiation [7], or patterns of haplotype homozygosity [8].

In population genomics, haplotype analysis relies on the principle that beneficial mutations confer survival or reproductive advantages in a specific environment [9]. Over generations, carriers of such alleles proliferate, increasing the frequency of linked neutral variants via genetic hitchhiking [10]. This process creates extended genomic regions with high linkage disequilibrium (LD) and reduced genetic diversity. Since recombination breaks down these haplotypes over time, recent selective sweeps are detectable as regions of extended haplotype homozygosity (EHH), characterized by unusually long, high-frequency haplotypes [11]. Methods based on this principle—including iHS, nSL, iHH12, and XP-EHH [12,13,14,15]—are now standard tools for detecting such selection signatures.

This study presents the first pilot analysis of the genomic architecture of the Salsk sheep breed using haplotype-based methods (iHS, nSL, iHH12, and XP-EHH) in comparison with four other breeds. It aims to evaluate the population’s genetic structure and identify distinctive adaptive features via a genome-wide scan for selection signatures involved in productivity and environmental adaptation.

## 2. Materials and Methods

### 2.1. Animals and Genotyping

Biological samples (ear notches) were collected from 96 Salsk sheep maintained at a stud farm in the Russian Federation. The sampling was performed by the breeding farm staff during standard zootechnical procedures that did not require any additional manipulation of the animals; therefore, separate approval from a bioethics committee was not required. Animals with close genetic relationships (kinship coefficient ≤ 0.125 according to breeding records) were excluded from the selection. The dataset included animals of both sexes: 20 rams (20%) and 76 ewes (80%). All animals were clinically healthy and aged between 2 and 5 years. Genotyping was performed using the high-density Illumina Ovine Infinium^®^ HD SNP BeadChip (Illumina Inc., San Diego, CA, USA). 

For comparative analyses, we used publicly available genotypes from four European breeds [16]: Mérinos d’Arles (MER, n = 18), Île-de-France (IDF, n = 23), Suffolk (SUF, n = 19), and Texel (TEX, n = 24). Salsk sheep (n = 96) were sampled at a stud farm in the Rostov region, Russia (continental steppe). MER originates from the Mediterranean/sub-Mediterranean south of France (fine wool), TEX from the North Sea basin with a temperate oceanic climate (meat), while IDF and SUF represent French/Western European commercial systems (meat/wool composite and meat). These geographic and production-ecology contrasts were intentionally leveraged to maximize discriminatory power for Salsk-specific adaptation signals.

### 2.2. Quality Control and Data Filtering

The data analysis was carried out on a Linux server with the following specifications: 128 GB of RAM and a 24-core processor. We merged the datasets and performed quality control using PLINK v1.9 [17]. The combined dataset was filtered using the following criteria: a single nucleotide polymorphism (SNP) call rate > 90% (--geno 0.1), an individual call rate > 90% (--mind 0.1), a minor allele frequency (MAF) > 5% (--maf 0.05), and a Hardy–Weinberg equilibrium (HWE) *p*-value > 10^−3^. Following quality control, 511,145 SNPs remained for subsequent analysis.

The average GenCall Score (GC) was 0.74, and the mean Genotype Call Rate was 0.984. All samples with GC values below 0.6 were excluded from the analysis. These summary values were calculated after the quality control filtering described above. These parameters meet the established quality standards for the Illumina Ovine Infinium^®^ HD BeadChip and confirm the high reliability of the genotyping data.

### 2.3. Population Structure and Genetic Diversity Analysis

We performed principal component analysis (PCA) to visualize genetic relationships between breeds using the snpgdsPCA function from the SNPRelate package in R. Pairwise fixation index (Fst) values between populations were calculated using the --fst option in PLINK v1.9. We assessed linkage disequilibrium (LD) decay for each breed by calculating the mean coefficient of determination (r^2^) between SNP pairs across varying physical distances.

### 2.4. Detection of Positive Selection Signatures

To identify genomic regions under selection, we used several haplotype-based statistical methods. We identified intrapopulation selection signatures using the integrated haplotype score (iHS), the number of segregating sites by length (nSL), and the integrated haplotype homozygosity pooled (iHH12) statistic, which is particularly sensitive to recent, strong selective sweeps. For interpopulation analysis, we used the cross-population extended haplotype homozygosity (XP-EHH) statistic, which identifies alleles approaching fixation in one population compared to another. All calculations were performed using Selscan v1.3.0 [18]. Raw scores were normalized within each chromosome, and we used the top 1% of the distribution as the threshold for identifying candidate selection signatures.

To reduce sampling imbalance, we performed randomized subsampling to obtain comparable group sizes (n ≈ 18–24 per breed) for the XP-EHH and Fst analyses; this procedure controls for unequal sample sizes and minimizes statistical bias. Randomization was implemented in R (v4.3.1).

### 2.5. Annotation of Candidate Genes

SNPs within the top 1% of selection signals were mapped to candidate genomic regions. We annotated these regions and identified the genes within them using the Ensembl Variant Effect Predictor (VEP) (https://www.ensembl.org/info/docs/tools/vep/index.html, accessed on 3 August 2025) against the *Ovis aries* reference genome assembly (ARS-UI_Ramb_v2.0). Subsequently, we conducted a literature review (PubMed, Google Scholar) to determine the functions of these candidate genes and their known associations with relevant biological processes and phenotypic traits in livestock.

## 3. Results and Discussion

### 3.1. Principal Component Analysis (PCA)

**Figure 1:**
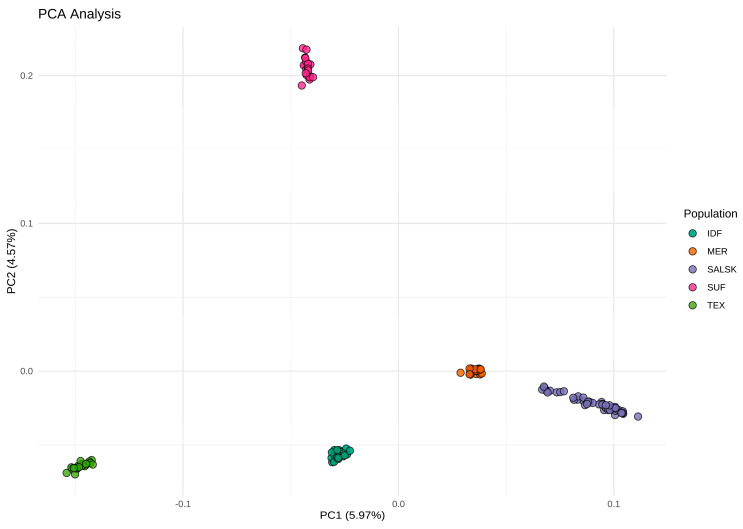
Principal component analysis (PCA) of genetic relationships among the Salsk sheep and four reference breeds. (The plot displays individual animals projected onto the first two principal components (PC1, 5.97% of variance; PC2, 4.57%). Each point is colored according to its breed: SALSK (Salsk); MER (Mérinos d’Arles); IDF (Île-de-France); SUF (Suffolk); and TEX (Texel).

We visualized the genetic structure and relationships among the populations using principal component analysis (PCA) (Figure 1). The results show distinct clusters corresponding to breed origin, which indicates significant genetic differentiation among the five populations studied.

The first two principal components, PC1 (5.97%) and PC2 (4.57%), accounted for a combined 10.54% of the total genetic variation and effectively separated the populations into distinct clusters. Subsequent components (PC3–PC5) explained an additional 7.3% of the total variance and did not reveal any further clustering or population substructure beyond that observed along PC1 and PC2. Notably, the Salsk and Mérinos d’Arles clusters are in close proximity on the plot. Both breeds cluster on the positive end of the PC1 axis, visually confirming their close genetic relationship, which is consistent with their shared Merino ancestry.

However, the Salsk cluster displays greater dispersion along the PC1 axis compared to the more compact Mérinos d’Arles cluster. This may indicate higher genetic diversity within the Salsk population. The reference meat breeds, Suffolk and Texel, formed distinct clusters, separated from each other and from the Merino-type breeds. Specifically, Suffolk occupies a unique position in the upper-right quadrant (high positive PC2 values), while Texel is situated in the lower-left quadrant. The Île-de-France (IDF) breed, which has Merino ancestry via the Rambouillet, is positioned intermediate to the Merino and meat breed clusters, yet forms its own distinct group.

Overall, the PCA shows that Salsk is a genetically distinct Merino-derived population positioned closest to Mérinos d’Arles (pairwise Fst = 0.04), while remaining clearly separated from the meat-type references. This provides a solid foundation for investigating the genomic signatures underlying their distinctive adaptive traits.

### 3.2. Linkage Disequilibrium (LD) Analysis

**Figure 2:**
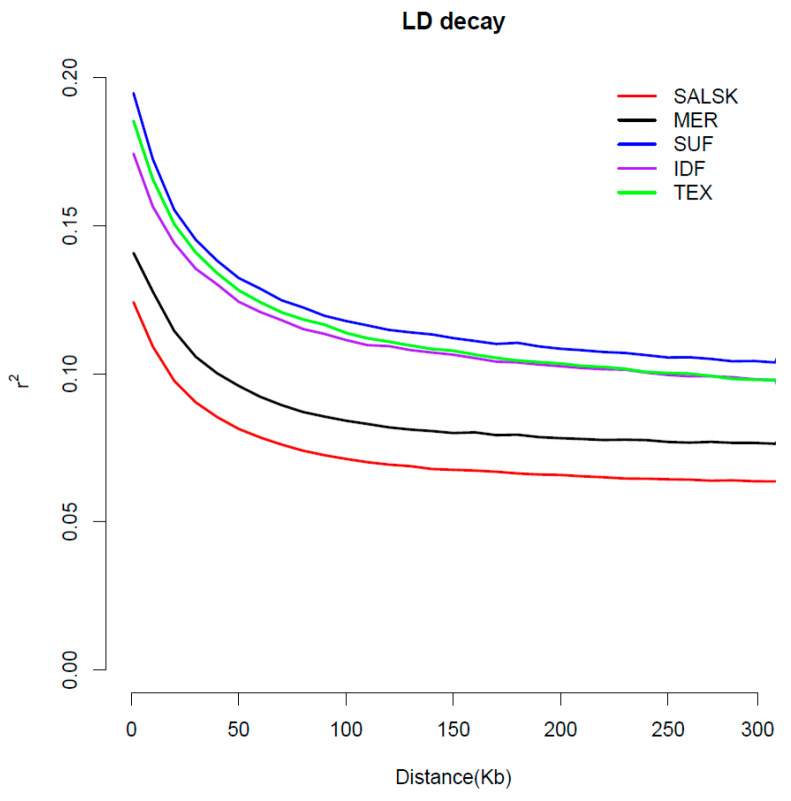
Comparative analysis of linkage disequilibrium (LD) decay in Salsk sheep and reference breeds. (The plot shows the mean linkage disequilibrium (r^2^) as a function of physical distance (kb) between SNP pairs. Each line represents a different breed: SALSK (Salsk); MER (Mérinos d’Arles); IDF (Île-de-France); SUF (Suffolk); and TEX (Texel).

Analysis of linkage disequilibrium (LD) decay revealed distinct patterns among the studied breeds, reflecting differences in their demographic histories and selection pressures (Figure 2).

The average genetic diversity estimates for the Salsk breed (SALSK) were as follows: expected heterozygosity (He) = 0.38, observed heterozygosity (Ho) = 0.36, and allelic richness = 1.92. The Salsk breed exhibited the lowest overall level of LD and the most rapid decay with increasing physical distance. This pattern is indicative of higher genetic diversity and a larger effective population size, likely stemming from its complex breed history involving multiple founder lines and less intense directional selection. Conversely, the Suffolk displayed the highest level of LD, which persisted over long genomic distances. This pattern is characteristic of breeds that have undergone intensive selection for specific traits (in this case, meat production) and/or experienced population bottlenecks, promoting the fixation of large haplotypes.

The Mérinos d’Arles, Île-de-France, and Texel breeds showed intermediate levels of LD decay. Notably, the decay curves for the Île-de-France and Texel meat breeds were nearly identical, positioned between the high LD of Suffolk and the more moderate LD of Mérinos d’Arles. These differing LD patterns suggest that the Salsk breed has retained substantial genetic diversity, whereas the specialized European breeds carry distinct genomic signatures of intensive artificial selection.

### 3.3. Genetic Differentiation Between Studied Sheep Breeds (Fst)

**Figure 3:**
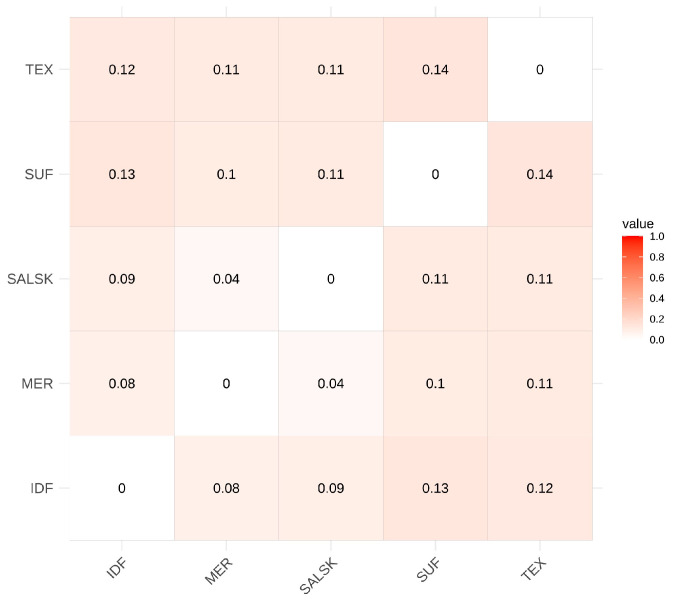
Genetic differentiation among the studied sheep breeds. (A heatmap of pairwise Fst values. The color intensity corresponds to the level of genetic differentiation, with darker shades of red indicating higher Fst values. Diagonal values are zero. Breed abbreviations: SALSK (Salsk); MER (Mérinos d’Arles); IDF (Île-de-France); SUF (Suffolk); and TEX (Texel)).

To quantify genetic differentiation among the populations, we calculated the pairwise fixation index (Fst) (Figure 3). The resulting Fst values ranged from 0.04 to 0.14, indicating moderate to significant genetic divergence among the breeds.

The lowest Fst value (0.04) was observed between the Salsk and Mérinos d’Arles breeds. This result quantitatively corroborates the close genetic relationship suggested by the PCA and is consistent with the Salsk breed’s development from a Merino gene pool. In contrast, the Salsk breed showed significant differentiation from the specialized meat breeds, with Fst values of 0.11 against both Suffolk and Texel, and 0.09 against Île-de-France.

Among the reference groups, the greatest genetic distances were observed between the meat breeds themselves: Suffolk and Texel (Fst = 0.14), and Suffolk and Île-de-France (Fst = 0.13). This finding underscores their distinct and independent selection histories. The differences between breed clusters observed in the PCA are consistent with the Fst calculations and reflect statistically significant population divergence. Thus, the Fst analysis strongly supports the clustering observed in the PCA and provides a robust foundation for the subsequent search for selection signatures.

### 3.4. Intra-Population Selection Signatures (iHS, nSL, iHH12)

**Figure 4:**
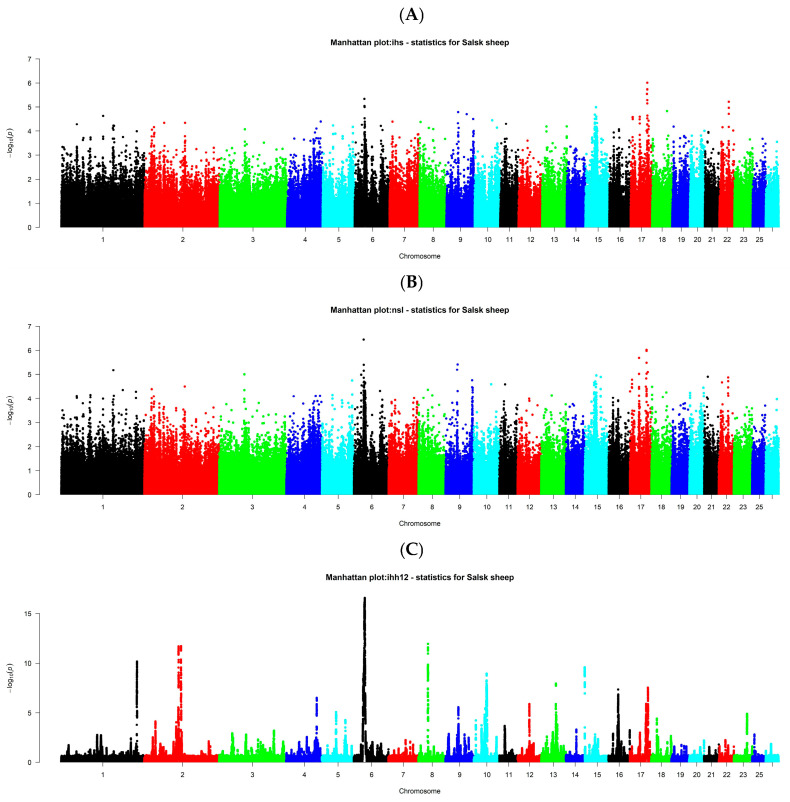
Signatures of selection in the Salsk sheep genome. Manhattan plots showing the results of a genome scan for signatures of positive selection using three statistical methods: (**A**) iHS; (**B**) nSL; and (**C**) iHH12. The x-axis represents the chromosomes, and the y-axis represents the level of statistical significance (−log10 *p*-value). High peaks indicate genomic regions presumably under selection.

To identify signatures of selection within the Salsk breed, we performed a haplotype-based analysis using the iHS, nSL, and iHH12 statistics (Figure 4).

These complementary methods detect genomic regions with extended haplotype homozygosity, which are indicative of recent or ongoing positive selection for advantageous alleles (Table 1).

The strongest selection signal, detected by all three methods, was located in the vicinity of the *CCSER1* gene. This gene is a key regulator of the cell cycle, cell growth, and fertility [19,20], positioning it as a prime candidate for selection on improved reproductive traits and growth rates in the Salsk breed. Strong signals, supported by both iHS and nSL, were also identified near the *SOX6*, *ACSL5*, and *CUX2* genes. The *SOX6* gene encodes a critical regulator of muscle, cartilage, and hair follicle development [21,22], suggesting concurrent selection for both meat and wool characteristics. The *ACSL5* gene encodes long-chain acyl-CoA synthetase 5, an enzyme involved in fatty acid metabolism [23,24], and selection on this gene is likely linked to enhanced metabolic efficiency, energy balance, and adaptation to the forage conditions of the steppe. The *CUX2* gene is involved in neural and epidermal development [25], potentially connecting it to adaptive responses as well as skin and wool development. Analysis of the remaining candidate genes reveals several key themes of selection in the Salsk breed, including growth and meat productivity; in addition to *SOX6*, signals near *GHR* (growth hormone receptor) [26] and *TRIM55* (a regulator of muscle mass) [27,28] further support this theme. Collectively, these signals point to targeted selection to improve precocity and muscle development.

Selection also appears to have targeted genes related to metabolism and energy adaptation. Besides *ACSL5*, these include *OXCT1* (involved in ketone body metabolism) [29] and *HECTD4* (linked to lipid metabolism) [30], underscoring the importance of efficient energy utilization for survival in the harsh steppe environment. Furthermore, signals near *CRH*, *DAPP1*, and *LRFN5* are likely associated with adaptation, stress resistance, and immunity. The CRH gene, encoding corticotropin-releasing hormone, is central to the neuroendocrine regulation of the stress response [31,32], while the *DAPP1* and *LRFN5* genes are involved in immune function [33,34,35,36,37] and may reflect selection for resistance to local pathogens.

Overall, the detected selection signatures point to complex, polygenic adaptation. The consistent identification of strong signals near *CCSER1*, *SOX6*, and *ACSL5* across multiple methods highlights them as targets of fundamental, long-term selection in the breed. In contrast, genes identified by only a single method may reflect more specific or recent adaptive events. These data suggest that the Salsk gene pool has been shaped by selection for dual-purpose (meat and wool) productivity, fertility, and high adaptive plasticity. The identified candidate genes provide valuable targets for further functional validation and for the strategic design of future breeding programs.

### 3.5. Interpopulation Signatures (XP-EHH)

**Figure 5:**
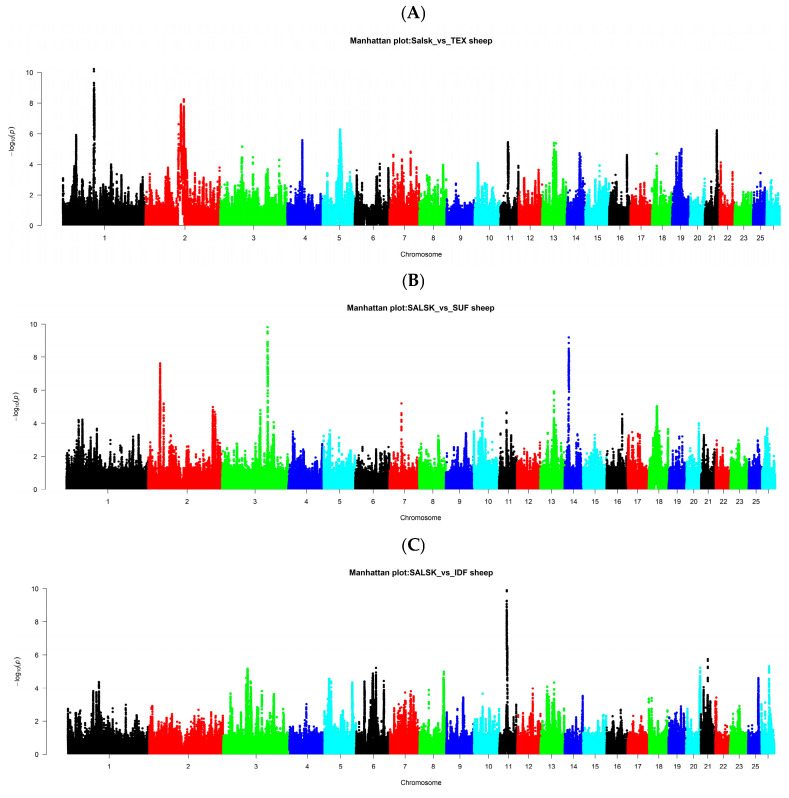
Analysis of selection signatures in the Salsk breed using the XP-EHH statistic. (Manhattan plots showing the results of the XP-EHH (Cross-Population Extended Haplotype Homozygosity) test in pairwise comparisons of the Salsk breed (SALSK) genome against other breeds: (**A**) vs. Texel (TEX); (**B**) vs. Suffolk (SUF); (**C**) vs. Île-de-France (IDF). The y-axis represents the statistical significance (−log10 *p*-value). High peaks indicate genomic regions presumably under positive selection in the Salsk breed relative to each of the reference breeds. Breed abbreviations: SALSK (Salsk); IDF (Île-de-France); SUF (Suffolk); and TEX (Texel)).

To identify genomic regions under positive selection specific to the Salsk breed, we applied the cross-population extended haplotype homozygosity (XP-EHH) method, comparing the Salsk population against each of the four European reference breeds. This approach detects loci where an allele has risen to high frequency or near fixation in a target population relative to a reference population, signaling divergent selective pressures. Our analysis focused on regions with high positive XP-EHH scores, as these indicate selection events in the Salsk lineage (Figure 5 and Supplementary Materials).

### 3.6. Salsk vs. Texel

The comparison against the Texel meat breed identified the most significant positive selection signal (XP-EHH) in the Salsk genome on chromosome 1 (Figure 5A). This signal corresponds to an extended haplotype block encompassing the interleukin-6 receptor (*IL6R*) gene. A dense cluster of over 80 SNPs with extremely high XP-EHH values in this region indicates that this haplotype is near fixation in the Salsk population but remains rare in Texel.

The biological significance of this signal likely involves two interrelated functions: immune adaptation and energy metabolism. As the receptor for the key cytokine IL-6, IL6R is central to immune responses and inflammation. Selection on this gene likely reflects the Salsk breed’s adaptation to the specific pathogen pressures and harsh climatic conditions of the Salsk steppes. This hypothesis is supported by recent studies where IL6R polymorphisms were associated with resistance to bacterial pneumonia in Barki sheep, another breed adapted to harsh environments [38]. Such intense selective pressure was likely absent in the Texel breed, which developed in a milder island climate.

This interpretation is reinforced by the pleiotropic roles of IL-6 in regulating energy metabolism and muscle growth, suggesting the signal may also be linked to metabolic adaptation. The functions of neighboring genes, such as *KCNN3* (involved in regulating muscle contraction and blood flow) [39] and PMVK (a key enzyme in cholesterol synthesis) [40], further support this hypothesis. Thus, the selected haplotype likely confers a complex pleiotropic advantage, enhancing both immune resilience and metabolic efficiency. These are critical adaptive traits for survival and productivity in the harsh environment of the Salsk steppes.

### 3.7. Salsk vs. Suffolk

Comparison with the highly specialized Suffolk meat breed revealed several significant selection signals indicating divergent evolutionary pathways (Figure 5B). A notable signal in the Salsk breed was a haplotype on chromosome 3 encompassing the *IRAK3* gene, a known negative regulator of innate immunity [41]. Selection on this haplotype likely represents an adaptive strategy for modulating the immune response, possibly preventing excessive inflammation under conditions of chronic stress or high pathogen load. This conclusion is bolstered by a signal near the *DOCK5* gene (chromosome 2), which is involved in immune cell migration. Critically, *DOCK5* was independently identified as a candidate for adaptation in Iranian sheep breeds adapted to similar harsh conditions [42], providing strong evidence for its fundamental role in sheep adaptation to environmental stressors.

A signal was also identified on chromosome 3 within the *WIF1* gene, an inhibitor of the Wnt-signaling pathway. The key role of Wnt signaling, and *WIF1* specifically, in skin and hair follicle development in sheep has been well-established [43,44]. This makes *WIF1* a prime candidate to explain selection for the sharply contrasting wool types between the breeds: the long, fine wool of Salsk sheep versus the short, coarse wool of Suffolk. Divergent selection on this gene could directly influence fiber density, length, and crimp. Furthermore, a secondary pleiotropic effect of selection on *WIF1* could be differences in metabolism, as Wnt signaling is also involved in adipogenesis. Thus, the allele selected for wool improvement in the Salsk breed may have also pleiotropically contributed to the fat deposition necessary for surviving harsh winters.

The most extensive selection signal in the Salsk breed was identified on chromosome 14, a large block indicative of a major selective sweep. This block harbors genes implicated in fundamental processes key to the Salsk breed’s divergent adaptation, including metabolic efficiency, cellular resilience, and muscle function. In the context of metabolism, a central candidate is the *ACSF3* gene, an acyl-CoA synthetase important for mitochondrial fatty acid metabolism. The importance of this gene family in livestock is highlighted by studies linking it to milk flavor in goats [45] and muscle fat deposition in pigs [46,47]. Selection on this gene in the Salsk breed was likely aimed at optimizing energy metabolism for the efficient use of sparse steppe forage.

Another key selection theme in this locus is cellular stability and stress adaptation, where the *FANCA* gene, a core component of the DNA repair system, plays a central role. Notably, *FANCA* has been repeatedly identified as a target of selection in locally adapted breeds of pigs and cattle [48,49,50,51], strongly suggesting its fundamental role in adaptation to adverse environmental conditions. For the Salsk breed, selection on *FANCA* may have conferred enhanced cellular resistance to stressors such as plant toxins, temperature extremes, or ultraviolet radiation, thereby improving overall survivability. Finally, selection signals in the *MYLK3* gene, encoding myosin light chain kinase, are likely associated with muscle tissue modifications. Studies in sheep have linked this gene to meat quality and other production traits [52,53]. For the Salsk breed, selection on *MYLK3* may have had a dual purpose: improving meat productivity while simultaneously enhancing endurance, possibly through optimizing cardiac muscle function.

### 3.8. Salsk vs. Île-de-France

Comparison with the Île-de-France breed revealed a distinct positive selection signal in the Salsk genome on chromosome 11 (Figure 5C). A cluster of genes in this region—including *NLRP1*, *RABEP1*, *ZFP3*, *KIF1C*, *MINK1*, *GLTPD2*, and *TM4SF5*—appears to represent a co-adapted complex implicated in resistance to infectious agents and environmental stressors. The key candidate gene here is *NLRP1*, which encodes a component of the inflammation that initiates inflammatory responses to pathogens and cellular stress. The importance of Nod-like receptors (*NLR*s) in sheep immunity has been established in studies of their expression in immune-competent organs [54,55,56,57]. We hypothesize that selection on the NLRP1 haplotype in the Salsk breed reflects adaptation to specific pathogen pressures within the steppe ecosystem. This interpretation is bolstered by the functions of adjacent genes, such as *RABEP1* (involved in intracellular transport and antigen presentation) [58], *MINK1* (a regulator of stress-response pathways) [59], and *TM4SF5* (a coordinator of immune responses) [60]. The locus also contains *KIF1C*, whose homologs have been linked to adaptation in other species [61], further strengthening the case for this region’s adaptive significance. Comparison with the genetically close Mérinos d’Arles (MER) breed revealed no significant differences indicative of divergent selection paths, which is consistent with the low overall Fst value.

To provide an integrated view of divergent selection, we summarized the top XP-EHH candidate loci across all pairwise contrasts and grouped them by functional class (immunity, metabolism/energy, growth/tissue development, wool traits). The consolidated list is presented in Table 2.

## 4. Discussion

PCA and pairwise Fst corroborate a close genetic proximity between Salsk and Mérinos d’Arles (Fst = 0.04), while the broad within-population spread and rapid LD decay in Salsk indicate preserved genetic diversity. These population-genomic patterns are consistent with prior reports on Merino and Merino-derived breeds. The close proximity between Salsk and Mérinos d’Arles in PCA and their low pairwise differentiation mirror the moderate within-Merino divergence described in large comparative panels of Merino lineages worldwide [15] and in regional surveys of European fine-wool breeds [13,16]. The LD profiles we observe—rapid decay in Salsk and elevated, long-range LD in Suffolk—also align with findings that locally adapted or less intensively selected populations retain faster LD decay and higher diversity, whereas specialized meat breeds show persistent LD due to strong directional selection and/or past bottlenecks [13,15]. At the same time, Salsk remains clearly separated from meat-type references (Suffolk, Texel), with Île-de-France intermediate, providing the population-genomic context for interpreting the selection signatures reported below. This genome-wide analysis of selection signatures reveals the genetic architecture of the Salsk sheep breed, shaped primarily by selection for adaptation to the harsh continental steppe environment. The identified genomic regions under selection indicate that the breed’s evolutionary trajectory favored enhanced overall resilience rather than narrow specialization in a limited set of production traits. The central driver of selection was adaptation to a complex of abiotic stressors, including high summer temperatures, seasonal droughts, and limited forage availability (Table 2).

Accordingly, the most significant selection signals were identified in genes controlling thermoregulation and energy metabolism. These include genes involved in fatty acid and ketone metabolism (*ACSL5*, *OXCT1*, *ACSF3*) and DNA repair (*FANCA*), which collectively point to selection for metabolic flexibility. This trait ensures efficient energy utilization and conservation, which is critical for survival under fluctuating resource conditions.

Another key direction of selection was the modulation of the immune system. Our analysis revealed signals in genes responsible for both the activation (*IL6R*, *NLRP1*) and negative regulation (*IRAK3*) of immune responses, suggesting the evolution of a balanced immune strategy. This fine-tuning allows the organism to effectively resist pathogens endemic to steppe ecosystems while avoiding excessive, energetically costly inflammatory reactions—a clear evolutionary advantage under constant pathogen pressure.

Taken together, the concordant signals from intra-population scans indicate polygenic adaptation with recurrent involvement of pathways related to immune modulation, energy metabolism, and tissue development. Signals near regulators linked to fertility and growth (e.g., CCSER1) were among the more consistent, although causal interpretation of individual variants requires further investigation and functional validation.

In the context of modern livestock production, the Salsk gene pool represents a valuable genetic resource. While intensive selection in commercial breeds has often reduced adaptive breadth, our population-genomic context (close genome-wide proximity to Mérinos d’Arles, significant yet moderate Fst versus meat breeds, and rapid LD decay in Salsk indicating preserved diversity; Figure 1, Figure 2 and Figure 3), together with the functional profile of selection signals (immune/stress and energy-metabolism pathways alongside muscle and wool regulators such as SOX6, GHR, and WIF1; Figure 4 and Figure 5, Table 2), supports parallel selection on both productivity and adaptation. This pattern is consistent with long-term breeding under continental steppe conditions (heat, seasonal drought, sparse forage), where resilience and dual-purpose performance are simultaneously favored. This makes the breed a valuable donor for introgressing adaptive alleles into the genomes of highly specialized breeds to enhance their environmental resilience. Such an approach is particularly relevant in the face of global climate change and the growing need for more sustainable livestock systems.

Of particular interest is the convergence in functional classes of selection signatures. In the study by Yousefi et al. [42] on Iranian meat and dairy breeds, genes under selection were related to immune response (e.g., IL23A, STAT2, DOCK5) and productivity (e.g., NPY, MYF5). Similar gene categories were identified in the Salsk breed, pointing to universal mechanisms of sheep adaptation to extreme environments.

The effectiveness of this study was enhanced by combining complementary methodological approaches. Intra-population statistics (iHS, nSL) were instrumental in identifying regions under ongoing or incomplete selective sweeps within the population. Conversely, inter-population analysis (XP-EHH) was most effective for detecting loci where alleles have reached near-fixation, reflecting long-term divergent selection events. The application of this comprehensive approach provided a complete and more reliable picture of the breed’s evolutionary history. Nevertheless, the identified signatures may partially reflect demographic processes. Therefore, more extensive studies using larger sample sizes and methods for reconstructing demographic history are required for definitive conclusions.

## 5. Conclusions

The conducted analysis revealed the unique genetic structure of the Salsk sheep breed. The results from PCA and fixation index (Fst) analyses confirmed their Merino origins while simultaneously demonstrating a clear genetic distinctness and a high level of within-population diversity. This is consistent with the evidence of rapid linkage disequilibrium (LD) decay and low genetic differentiation identified by Mérinos d’Arles (Fst = 0.04). Based on intra- and interpopulation scans, we identified >40 candidate regions. A substantial subset was supported by at least two methods and mapped to genes involved in responses to climatic stress and immunoregulation (IL6R, NLRP1), energy metabolism (ACSL5, OXCT1, ACSF3), and reproductive processes (CCSER1, SOX6, GHR). These results provide the first comprehensive description of the genomic architecture of the Salsk breed and elucidate the mechanisms underlying their high resilience. Collectively, our data characterizes the Salsk sheep as a valuable reservoir of adaptive alleles, holding strategic importance for future breeding programs aimed at enhancing the sustainability of sheep farming.

## Figures and Tables

**Table 1:** Candidate genes identified as selection signatures in Salsk sheep.

Gene	Functions	iHS	nSL	iHH12
*CCSER1*	Cell cycle, division; growth, fertility	✓	✓	✓
*ACSL5*	Lipid metabolism, energy balance	✓	✓	
*CUX2*	Nervous system development, skin/wool	✓	✓	
*SOX6*	Muscle and cartilage differentiation; skeletal development	✓	✓	
*LRFN5*	Synaptic function, nervous system	✓		
*RASSF10*	Cell cycle, growth regulation (Ras pathway)		✓	
*WNT11*	Wnt signaling; embryogenesis, reproduction		✓	
*DAPP1*	Immune response (B-cells)		✓	
*ATP6V0D2*	Bone metabolism, osteoclasts		✓	
*ARHGEF4*	Wnt/adhesion (RhoGEF); cell migration		✓	
*TRIM24*	Transcriptional regulation, differentiation			✓
*MMRN1*	Extracellular matrix, hemostasis; growth, fattening			✓
*CRH*	Stress hormone, neuroendocrine regulation			✓
*TRIM55*	Muscle protein (MuRF2); muscle mass regulation			✓
*OXCT1*	Ketone metabolism; energy adaptation			✓
*GHR*	Growth hormone receptor; growth, lactation			✓
*CIT*	Cytokinesis, neurodevelopment			✓
*HECTD4*	Ubiquitin ligase; lipid metabolism			✓
*SLC8B1*	Ion transport; heart rhythm			✓

A checkmark (✓) indicates that the gene was detected within the top 1% of signals for the corresponding selection-scan method (iHS, nSL, or iHH12).

**Table 2:** Candidate genes under positive selection in the Salsk breed identified by XP-EHH analysis.

Gene	Salsk Breed—Texel	Salsk Breed—Suffolk	Salsk Breed—Île-De-France
Immune Response and Disease Resistance			
*IL6R*	✓		
*IRAK3*		✓	
*DOCK5*		✓	
*NLRP1*			✓
*RABEP1*			✓
*TM4SF5*			✓
*FANCA*		✓	
*MINK1*			✓
*KIF1C*			✓
Metabolism and Energy Exchange			
*IL6R*	✓		
*PMVK*		✓	
*WIF1*		✓	
*ACSF3*		✓	
Growth, Tissue Development, and Productivity			
*KCNN3*	✓		
*MYLK3*		✓	
Wool development and pigmentation			
*WIF1*		✓	

A checkmark (✓) indicates that the gene was detected within the top 1% of signals for the corresponding selection-scan method (iHS, nSL, or iHH12).

## Data Availability

The original contributions presented in this study are included in the article/Supplementary Materials. Further inquiries can be directed to the corresponding authors.

## Supplementary Materials

The following supporting information can be downloaded at https://www.mdpi.com/article/10.3390/biology14111620/s1. File S1: Genetic variants.
